# TECHNOLOGY PREFERENCES AMONG MILD COGNITIVE IMPAIRMENT (MCI) DYADS LIVING IN A MEMORY CARE DESERT

**DOI:** 10.1093/geroni/igae098.3732

**Published:** 2024-12-31

**Authors:** Michael McCarthy, Eric Cerino, Megan McCoy, Margarita Martinez, Thomasina Seaton, Amanda Goldtooth, Raechel Livingston, Rasheera Dopson

**Affiliations:** Northern Arizona University, Flagstaff, Arizona, United States; Northern Arizona University, Flagstaff, Arizona, United States; Northern Arizona University, Northern Arizona University, Arizona, United States; Northern Arizona University, Northern Arizona University, Arizona, United States; Northern Arizona University, Flagstaff, Arizona, United States; Northern Arizona University, Flagstaff, Arizona, United States; Northern Arizona University, Flagstaff, Arizona, United States; Northern Arizona University, Flagtsaff, Arizona, United States

## Abstract

Digital health technologies hold promise for early detection and support of individuals and family members contending with Mild Cognitive Impairment (MCI), which may be an early step in the progression toward dementia. These technologies may be especially relevant for care dyads living in “memory care deserts” who are often isolated, under-resourced, experience geographic barriers to care, and lack access to health information and services. The aim of this study is to explore technology preferences of MCI care dyads participating in the Northern Arizona Memory Study in order to better understand the feasibility and acceptability of these technologies for intervention development and delivery. We used a mixed methods approach with semi-structured interviews conducted with 11 dyads comprised of an older adult with MCI and their caregiver (n=22). These data were combined with survey data about participant knowledge and use of technologies, as well as their preferences for mode of delivery for interventions (e.g., interactive website with professional coaching, text messages with peers dealing with memory loss). Qualitative data suggest that there is general interest in technology among MCI care dyads. However, comfort levels with specific technologies vary due to demographic, social, economic, personal, and structural factors. Survey data corroborate these findings with most participants indicating agreement about the usefulness of technologies for MCI detection and support, as well as access to and knowledge of these technologies. Researchers may benefit from these insights as they develop technology-based supportive interventions and resources for underserved MCI care dyads.

